# Electrochemical Sex Determination of Dioecious Plants Using Polydopamine-Functionalized Graphene Sheets

**DOI:** 10.3389/fchem.2020.00092

**Published:** 2020-03-06

**Authors:** Li Fu, Qiao Wang, Mingjun Zhang, Yuhong Zheng, Mengyao Wu, Ziyi Lan, Jiansong Pu, Huaiwei Zhang, Fei Chen, Weitao Su, Jinhong Yu, Cheng-Te Lin

**Affiliations:** ^1^College of Materials and Environmental Engineering, Hangzhou Dianzi University, Hangzhou, China; ^2^Jiangsu Key Laboratory for the Research and Utilization of Plant Resources, Institute of Botany, Jiangsu Province and Chinese Academy of Sciences, Nanjing, China; ^3^Institute of Botany, Jiangsu Province and Chinese Academy of Sciences, Nanjing Botanical Garden, Memorial Sun Yat-Sen, Nanjing, China; ^4^Key Laboratory of Marine New Materials and Related Technology, Zhejiang Key Laboratory of Marine Materials and Protection Technology, Ningbo Institute of Material Technology and Engineering, Chinese Academy of Sciences, Ningbo, China

**Keywords:** electrochemistry, polydopamine, graphene, dioecious plant, sex determination

## Abstract

The rapid identification of sex has potential uses involving dioecious commercial plants. In this work, we first propose a rapid electrochemical analysis method for plant sex determination using the signal difference generated by the electrochemically active substances in plant tissue. Polydopamine-functionalized graphene was wrapped around plant tissue. The introduction of polydopamine-functionalized graphene could solve the problem of the instability of plant tissue immobilization and enhance the electrochemical signals from plant tissue. *Taxus* × *media, Dioscorea zingiberensis*, and *Dioscorea bulbifera* were deliberately selected as dioecious plant models due to their pharmaceutical applications. The sex of the plant was not obvious after simply comparing the electrochemical voltammograms. Scatter patterns and 3D surface patterns were generated based on the voltammograms recorded after different solvent extractions. Sex determination was successfully achieved by pattern recognition.

## Introduction

Natural angiosperms are mostly monoecious, and dioecious plants only account for 6% of angiosperms. Dioecious plants play an important role in the study of sex chromosome evolution. Plants of different sexes often have different economic values. If the seeds and fruits of plants are the economic target, a large number of female plants are required. In contrast, in the case of green trees that are mainly vegetative, male plants sometimes have higher economic value. Therefore, studying the sex identification of dioecious plants is of great significance, both in theory and in practice. As early as the early 1950's, the Polish scholar Bugala first identified the sex of *Populus tremula* by leaf color (Bugala, 1951).

The identification of plant sex using physiological and biochemical differences is common technique. This method is mainly used to identify plant sex by analyzing the activity of certain enzymes, secondary metabolites, and endogenous hormone levels in plants. For example, Jaiswal et al. (1984) studied the acid and alkaline phosphatase activities of asexual and reproductive tissues of *Carica papaya* and found that the enzyme activity in males was higher than that in females. Although physiological and biochemical indicators can be used to identify the sex of dioecious plants under certain circumstances, the determination of these indicators is susceptible to environmental factors and different stages of plant growth. Chromosome morphological characteristics are also important indicators for the identification of male and female sex. However, the identification of the sex of dioecious plants by karyotype is only effective for species with distinct atypical chromosomes. This method has certain limitations for some species that do not have significant sex chromosomes or that have small differences in sex chromosomes. The identification of plant sex uses Jaiswal et al. (1984) and Kim et al. (2008). Enzymes are the direct product of gene expression. The organ tissue specificity of the isozyme spectrum also reflects the specificity of gene expression. Currently, isozyme studies related to sex differentiation are mostly focused on the peroxidase isoenzyme in male and female organs. Due to differences in varieties and tissue specificity of isoenzymes, the representativeness and conformity of the sex identification of a certain plant cannot be achieved in the early growth stage (Obbard et al., 2006; Sharma et al., 2010). The above-mentioned methods all require lab-based analytical technology. However, sex determination of dioecious plants is commonly required in the field. Therefore, the development of a quick and portable analytical method for plant sex determination is very important in many fields, such as horticulture and plant pharmacological science.

Electrochemical analysis of plants is an application based on solid-state electroanalytical chemistry. Due to the presence of a variety of electrochemically active species in plants, plant tissues immobilized on the surface of the electrode produce electrochemical signals during the voltammetric scan. These signals reflect the type and amount of electrochemically active species in the plant tissue. The difference in electrochemically active substances is partially caused by the difference in secondary metabolites and isozymes described above. Therefore, the variability of electrochemically active substances also has considerable potential for the sex recognition of dioecious plants.

Doménech-Carbó et al. have been conducting a series of pioneering works on the electrochemical analysis of plants since 2015 (Doménech-Carbó et al., 2015, 2017, 2018; Ortiz-Miranda et al., 2016; Cebrián-Torrejón et al., 2017). We further introduced a graphene-wrapping process during plant tissue immobilization to enhance the electrochemical signal (Fu et al., 2018a,b). However, we found that the stability of the graphene-wrapped plant tissue needs to be further improved since the plant tissue could fall off during the addition of electrolytes.

Surface modification of materials has always been one of the hotspots of material science research. Mussels can attach to virtually any type of organic or inorganic material; this universal adhesive property is attributed to the repeated 3,4-dihydroxy-L-phenylalanine (DOPA) motif in mussel foot proteins. Inspired by this motif, small molecules containing catecholamine functional groups, such as dopamine, can be used to mimic mussel adhesion. In the study of this adhesion mechanism, it was found that under aqueous conditions, dopamine can undergo oxidative polymerization on a series of solid materials such as polymers, metals, ceramics, glass, and wood and form a thin, strong, viscous composite layer on the surface (Fu et al., 2015a,b, 2016; Qu et al., 2016; Ryu et al., 2018). Therefore, we assume that the introduction of polydopamine-functionalized graphene to plant tissue could solve the immobilization stability problem. In this work, we attempt to use polydopamine-functionalized graphene to wrap dioecious plants and quickly determine their sexes using an electrochemical voltammogram. *Taxus* × *media, Dioscorea zingiberensis*, and *Dioscorea bulbifera* were selected as dioecious plant models due to their economic value.

## Experimental Methods

### Synthesis of Polydopamine-Functionalized Graphene

In a typical synthesis of polydopamine-functionalized graphene, 10 mL of graphene ethanol dispersion (3 mg/mL) was first dispersed in Tris-buffer (30 mL, 10 mM, pH 8.5) and sonicated for 1 h. After the addition of 5 mg of dopamine, the mixture solution was stirred for 24 h at room temperature. The resulting solution was filtered using a 200-nm pore size filter paper. The solid part was collected and re-dispersed into an ethanol solution (0.5 mg/mL) to yield polydopamine-functionalized graphene.

### Plant Tissue Treatment

The leaf and stem of *Taxus* × *media*, leaf of *Dioscorea zingiberensis*, and leaf and bulbil of *Dioscorea bulbifera* were collected from Nanjing Botanic Garden (Jiangsu, China) in September 2018. All plant tissues were stored at −20°C before analysis. Water, ethanol, and N,N-dimethylformamide (DMF) were used as solvents for plant tissue extraction. Typically, 2 mg of chopped plant tissue was introduced into 0.5 mL of solvent and sonicated for 5 min. Then, 10 μL of polydopamine-functionalized graphene dispersion was added to the mixture and sonicated for another 5 min for tissue wrapping.

### Electrochemical Determination

All electrochemical determination processes were carried out using a portable CHI410C electrochemical workstation. A commercial screen-printed electrode (SPE, Nanjing Youyun Technology Co., Ltd.) with a three-electrode system was used for electrochemical determinations. Typically, 1 μL of the abovementioned plant tissue dispersion was drop-coated on the working electrode surface of the SPE and dried at room temperature. Then, 40 μL of 0.1 M PBS was dropped on the SPE surface to cover the three electrodes. A differential pulse voltammetry (DPV) scan was then conducted to record the electrochemical voltammogram.

### Pattern generation

For 2D pattern generation, the normalized current value recorded after two solvents extractions were used as the x and y axes. The normalized voltammetric data recorded after methanol, glycol, and water extractions have been used for 3D surface pattern (coupled with Plotly online mode) and color tape generation (grid package).

### Results and Discussion

**Figure 1:**
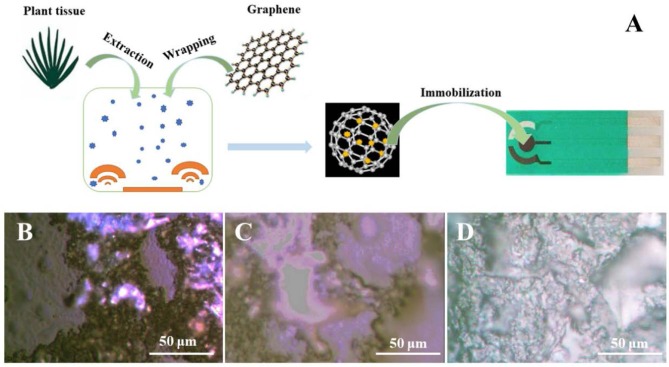
**(A)** Scheme of electroanalysis of polydopamine-functionalized graphene-wrapped plant tissue. Optical images of **(B)** plant tissue **(C)** and polydopamine-functionalized graphene-wrapped plant tissue **(D)**.

The overall flowchart for electrochemically identifying plant sex is shown in Figure 1A. As shown in the scheme, the plant tissue first dissolves a portion of the compounds during a simple extraction process. These compounds are adsorbed by polydopamine-functionalized graphene during the wrapping process and are immobilized on the SPE. Polydopamine-functionalized graphene not only stably anchors plant tissues on the surface of the electrode but also enhances the electrochemical signal expression of these substances by adsorption. It can be seen from the optical microscope image that plant tissue without the wrapping process cannot be uniformly immobilized on the electrode surface (Figure 1B). In contrast, the plant tissue wrapped by polydopamine-functionalized graphene has a very good film-forming property on the surface of the electrode (Figure 1D), which can effectively immobilize the plant tissue on the electrode surface without falling off. In addition, we also added 2 mg dopamine during the graphene sheets surface functionalization (Figure 1C). The deficiency of dopamine could affect immobilization performance.

**Figure 2:**
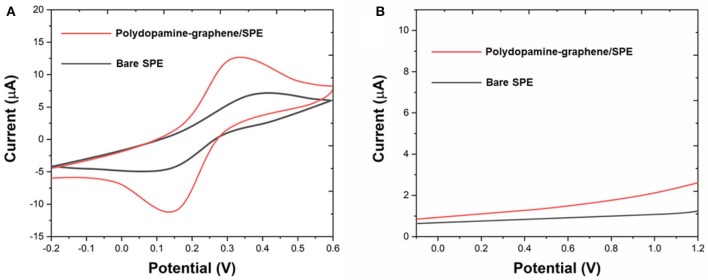
**(A)** CV of bare SPE and polydopamine-graphene/SPE in 5 mM [Fe(CN)_6_]^3−/4−^. **(B)** DPV of bare SPE and polydopamine-graphene/SPE in 0.1 M PBS.

We compared the electroactive surface areas (ESA) of SPE and polydopamine-functionalized graphene modified SPE. [Fe(CN)_6_]^3−^/^4−^ was used as an electrochemical probe. As shown in Figure 2A, cyclic voltammetry results showed that the peak–peak separation of bare SPE and polydopamine-functionalized graphene modified SPE were 147.51 and 279.23 mV, respectively. ESA of the bare SPE and polydopamine-functionalized graphene modified SPE can be calculated according to the Randles–Sevcik equation. The ESA values of the bare SPE and polydopamine-functionalized graphene modified SPE were calculated to be 0.0276 and 0.0795, respectively. Therefore, lamellar polydopamine-functionalized graphene sheets can provide a large effective area for electrochemical reaction. We further conducted the DPVs of bare SPE and polydopamine-functionalized graphene modified SPE 0.1 M PBS (Figure 2B). As shown in the figure, both electrodes showed no interference peaks, suggesting the electrode and its surface modification have no interference toward the electrochemical fingerprint recording.

**Figure 3:**
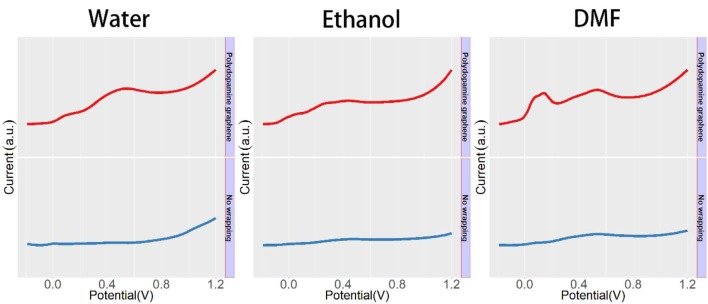
DPV curves of male *Taxus* × *media* leaf with and without functionalized graphene wrapping process.

The effect of the polydopamine-functionalized graphene wrapping process on the electrochemical signals of male *Taxus* × *media* leaf is shown in Figure 3. The male *Taxus* × *media* leaf without the wrapping process exhibits several peaks between −0.2 and 1.2 V, corresponding to some bioactive components, such as anthocyanins, flavonols, procyanidins, and phenolic acids (Romano et al., 2000; Emerenciano et al., 2001; Jensen et al., 2002; Dijkman and Kromkamp, 2006; Yin et al., 2018). However, these electrochemical signals are not very obvious, because the leathery leaves of *Taxus* × *media* make it difficult to dissolve a large amount of electrochemically active compounds in a simple extraction process. In contrast, polydopamine-functionalized graphene *Taxus* × *media* leaf tissue exhibits more pronounced electrochemical signals. The increase in these electrochemical signals is due to the large specific surface area of graphene, which adsorbs a large amount of dissolved compounds during the wrapping process. In addition, polydopamine-functionalized graphene wrapping can effectively immobilize leaf tissue on the electrode surface and provide a complete conductive network, allowing more plant tissue to participate in the electrochemical reaction. It can be seen from the figure that the participation of polydopamine-functionalized graphene increases the intensity of the original peak without causing an additional electrochemical peak, suggesting that the introduction of polydopamine-functionalized graphene has no influence on the electrochemical profile expression of the plant tissue. The current value of the voltammogram can be changed using different ratio combinations of polydopamine-functionalized graphene and plant tissue. However, the normalized voltammogram showed excellent stability unless the deficiency of polydopamine-functionalized graphene was used for plant tissue wrapping. Based on the above results, we further conducted a study of the sex determination of dioecious plants.

**Figure 4:**
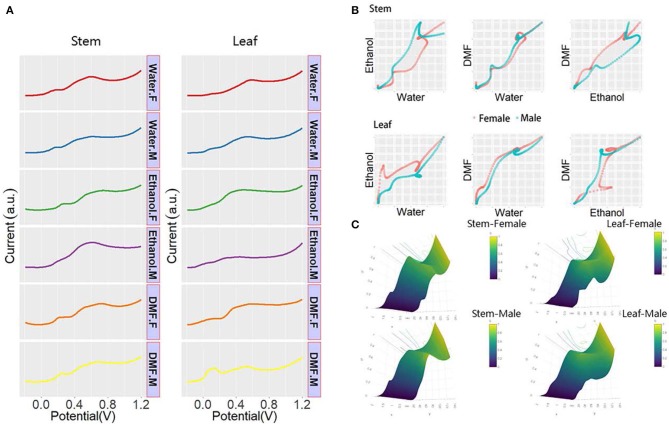
**(A)** DPV curves of plant tissue of *Taxus* × *media* recorded after water, ethanol, and DMF extractions. **(B)** Scatter patterns of male and female *Taxus* × *media* generated using DPV data from water and ethanol. **(C)** 3D surface patterns of male and female *Taxus* × *media*.

*Taxus* × *media* is a typical species of dioecious plant that is a natural hybrid with *Taxus baccata* as the male parent and *Taxus cuspidata* as the female parent. *Taxus* × *media* has the advantages of developed lateral roots, fast growth, pruning resistance, and strong adaptability to the environment. The anticancer active substance paclitaxel can be extracted from the whole plant (Furmanowa and Syklowska-Baranek, 2000; Zhang et al., 2000; Yang et al., 2018). *Taxus* × *media* can be a good raw material for the extraction of paclitaxel, which is of great significance to the medical field. Some studies have shown that the paclitaxel level varies in different sexes of *Taxus* × *media* (Nadeem et al., 2002; Lansky et al., 2005; Wang et al., 2006). Therefore, sex determination for *Taxus* × *media* is necessary at the seedling stage. Figure 4A shows all DPV profiles recorded in the leaf and stem of both sexes. As shown in the figure, the plant tissue of *Taxus* × *media* showed different electrochemical voltammograms after extraction in different solvents, indicating that solvent selection can significantly affect the extraction of electrochemically active compounds. Thus, the electrochemical signal obtained under different solvent extractions can represent a portion of the electrochemically active species in the plant tissue. We can see that the DPV curves of the stem showed more signals than the DPV curves of the leaf, indicating that the stem contains more electrochemically active substances or that the leathery leaves reduce the dissolution of the material. Although we can find that there are some differences in the DPV profiles of males and females of *Taxus* × *media*, it is still very difficult to identify sex directly through the DPV profiles. The differences in these signals can be combined into patterns of different dimensions for identifying the sex of the *Taxus* × *media*. As shown in Figure 4B, the DPV curves recorded after water and ethanol extractions were normalized and placed on a coordinate system to obtain a two-dimensional scatter pattern. In this scatter pattern, we can clearly see the difference between male and female *Taxus* × *media*. The 3D surface patterns of both male and female *Taxus* × *media* were generated from DPV data recorded after water, ethanol, and DMF extractions. These patterns can be used for sex recognition of *Taxus* × *media*. The pattern from the stem of *Taxus* × *media* showed more obvious sex differences than the pattern from the leaf. Therefore, the electrochemical profile of *Taxus* × *media* stem can be used for sex determination.

*Dioscorea bulbifera* is a worldwide species that has an extremely complex breeding system. Sexual reproduction and asexual reproduction coexist in the wild, so many species are natural polyploids. The sex of *Dioscorea* may be determined by sex chromosomes (Martin, 1966). The chromosomes of *Dioscorea* are mostly punctate or short and rod-shaped, with lengths normally <5 μm. Therefore, it is impossible to clearly distinguish the *X* or *Y* chromosomes by using the chromosomal tabletting technique.

**Figure 5:**
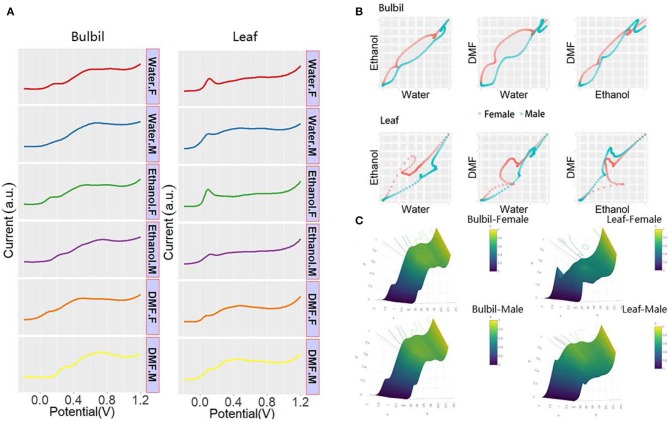
**(A)** DPV curves of plant tissue of *Dioscorea bulbifera* recorded after water, ethanol, and DMF extractions. **(B)** Scatter patterns of male and female *Dioscorea bulbifera* generated using DPV data from water and ethanol. **(C)** 3D surface patterns of male and female *Dioscorea bulbifera*.

It is difficult to identify the sex of *Dioscorea* using chromosome morphology. Therefore, the leaf and bulbil of *Dioscorea bulbifera* were collected for electrochemical sex determination. Figure 5A shows the DPV profiles of the plant tissue of *Dioscorea bulbifera*. As shown in the figure, the different sexes of *Dioscorea bulbifera* show less distinctiveness than the sexes of *Taxus* × *media*. DPV profiles cannot be directly used to distinguish sexes of *Dioscorea bulbifera*. Therefore, pattern recognition is required for successful determination. Figures 5B,C shows the 2D scatter pattern and 3D-surface pattern based on normalized data. Differences between the male and female *Dioscorea bulbifera* can be observed. Comparing the patterns shown from leaf and bulbil, the electrochemical profile of the *Dioscorea bulbifera* bulbil is more suitable for use in sex determination.

**Figure 6:**
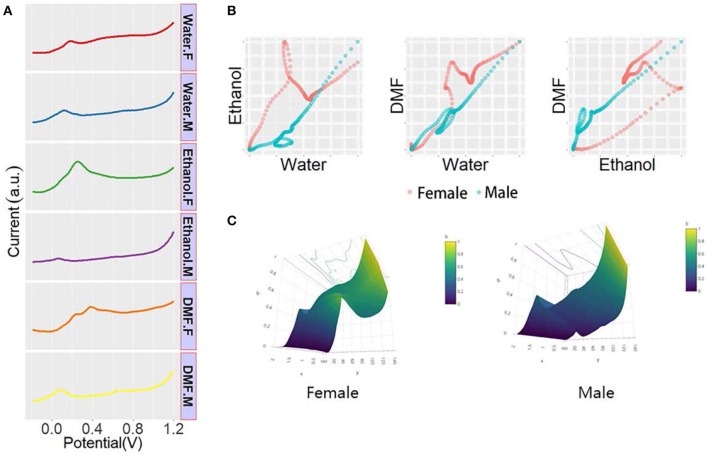
**(A)** DPV curves of plant tissue of *Dioscorea zingiberensis* recorded after water, ethanol, and DMF extractions. **(B)** Scatter patterns of male and female *Dioscorea zingiberensis* generated using DPV data from water and ethanol. **(C)** 3D surface patterns of male and female *Dioscorea zingiberensis*.

*Dioscorea zingiberensis* is another species of *Dioscorea* that only grows in China. *Dioscorea zingiberensis* has important medicinal value, and its rhizome, saponin, is an important starting material for synthetic contraceptives and steroid hormones, which play an important role in the pharmaceutical industry (Yang et al., 2016; Xiao et al., 2017). In this study, males and females of *Dioscorea zingiberensis* were deliberately selected for study. As shown in Figure 6A, the DPV curves show differences between sexes of *Dioscorea zingiberensis* recorded after water, ethanol, and DMF extractions. Scatter patterns (Figure 6B) and 3D surface patterns (Figure 6C) were generated using the aforementioned methods. As shown in the figure, the sexes of *Dioscorea zingiberensis* have been successfully determined using the proposed electrochemical method.

We further conducted MANOVA tests for our data. The p values of the variables (normalized DPV currents) recorded for *Taxus* × *media* (stem and leaf), *Dioscorea bulbifera* (bulbil and leaf), and *Dioscorea zingiberensis* (leaf) with the same sex were all larger than 0.05, indicating no significant differences. However, when comparing different sex, the p values were 3.7e^−07^, 4.3e^−07^, 6.1e^−06^, 6.0.7e^−07^, and 2.2e^−07^ for *Taxus* × *media* stem, *Taxus* × *media* leaf, *Dioscorea bulbifera* bulbil, *Dioscorea bulbifera* leaf, and *Dioscorea zingiberensis* leaf, respectively. This result suggests significant difference. Therefore, the differences of electrochemical fingerprints in a species with different sex are much larger than the same species.

## Conclusion

In conclusion, polydopamine-functionalized graphene could overcome the instability of plant tissue immobilization. In addition, the high surface area of the graphene could adsorb the electroactive compounds extracted during sonication and subsequently enhance the electrochemical signals. Sex determination for *Taxus* × *media, Dioscorea zingiberensis*, and *Dioscorea bulbifera* was successfully achieved using pattern recognition based on the voltammograms recorded during the electrochemical scan.

## Data Availability Statement

The raw data supporting the conclusions of this article will be made available by the authors, without undue reservation, to any qualified researcher.

## Author Contributions

LF, C-TL, WS, and YZ contributed conception and design of the study. QW, MZ, ZL, and JP conducted electrochemical experiments. MW, HZ, and FC performed the statistical analysis. LF and JY wrote the manuscript. All authors contributed to manuscript revision, read, and approved the submitted version.

### Conflict of Interest

The authors declare that the research was conducted in the absence of any commercial or financial relationships that could be construed as a potential conflict of interest.

## References

[B1] Bugala W. (1951). Sex determination of poplars from the color of leaves. Forestry Abstr. 52, 13–16.

[B2] Cebrián-Torrejón G. Doménech-Carbó A. Figadère B. Poupon E. Fournet A. (2017). Phytoelectrochemical analysis of *Zanthoxylum chiloperone*. Phytochem. Anal. 28, 171–175. 10.1002/pca.2657 27995663

[B3] Dijkman N. A. Kromkamp J. C. (2006). Phospholipid-derived fatty acids as chemotaxonomic markers for phytoplankton: application for inferring phytoplankton composition. Mar. Ecol. Prog. Ser. 324, 113–125. 10.3354/meps324113

[B4] Doménech-Carbó A. Ibars A. M. Prieto-Mossi J. Estrelles E. Doménech-Carbó M. T. Ortiz-Miranda A. S. (2017). Access to phylogeny from voltammetric fingerprints of seeds: the *Asparagus* case. Electroanalysis 29, 643–650. 10.1002/elan.201600588

[B5] Doménech-Carbó A. Ibars A. M. Prieto-Mossi J. Estrelles E. Scholz F. Cebrián-Torrejón G. (2015). Electrochemistry-based chemotaxonomy in plants using the voltammetry of microparticles methodology. New J. Chem. 39, 7421–7428. 10.1039/C5NJ01233C

[B6] Doménech-Carbó A. Montoya N. Soriano P. Estrelles E. (2018). An electrochemical analysis suggests role of gynodioecy in adaptation to stress in *Cortaderia selloana*. Curr. Plant Biol. 16, 9–14. 10.1016/j.cpb.2018.08.001

[B7] Emerenciano V. de P. Militão J. Campos C. Romoff P. Kaplan M. A. C.. (2001). Flavonoids as chemotaxonomic markers for *Asteraceae*. Biochem. Syst. Ecol. 29, 947–957. 10.1016/S0305-1978(01)00033-3 11445295

[B8] Fu J. Chen Z. Wang M. Liu S. Zhang J. Zhang J. (2015a). Adsorption of methylene blue by a high-efficiency adsorbent (polydopamine microspheres): kinetics, isotherm, thermodynamics, and mechanism analysis. Chem. Eng. J. 259, 53–61. 10.1016/j.cej.2014.07.101

[B9] Fu L. Lai G. Jia B. Yu A. (2015b). Preparation and electrocatalytic properties of polydopamine functionalized reduced graphene oxide-silver nanocomposites. Electrocatalysis 6, 72–76. 10.1007/s12678-014-0219-9

[B10] Fu L. Lai G. Zhu D. Jia B. Malherbe F. Yu A. (2016). Advanced catalytic and electrocatalytic performances of polydopamine-functionalized reduced graphene oxide-palladium nanocomposites. Chem. Cat. Chem. 8, 2975–2980. 10.1002/cctc.201600532

[B11] Fu L. Zheng Y. Zhang P. Zhang H. Zhuang W. Zhang H.. (2018a). Enhanced electrochemical voltammetric fingerprints for plant taxonomic sensing. Biosens. Bioelectron. 120, 102–107. 10.1016/j.bios.2018.08.052 30172233

[B12] Fu L. Zheng Y. Zhang P. Zhu J. Zhang H. Zhang L. (2018b). Embedding leaf tissue in graphene ink to improve signals in electrochemistry-based chemotaxonomy. Electrochem. Commun. 92, 39–42. 10.1016/j.elecom.2018.05.018

[B13] Furmanowa M. Syklowska-Baranek K. (2000). Hairy root cultures of taxus × media var. hicksii rehd. as a new source of paclitaxel and 10-deacetylbaccatin III. Biotechnol. Lett. 22, 683–686. 10.1023/A:1005683619355

[B14] Jaiswal V. Narayan P. Lal M. (1984). Activities of acid and alkaline phosphatases in relation to sex differentiation in *Carica papaya L*. Biochem. Physiol. Pflanz. 179, 799–801. 10.1016/S0015-3796(84)80009-8

[B15] Jensen S. R. Franzyk H. Wallander E. (2002). Chemotaxonomy of the Oleaceae: iridoids as taxonomic markers. Phytochemistry 60, 213–231. 10.1016/S0031-9422(02)00102-4 12031440

[B16] Kim S. Y. Kim C. S. Lee J. K. Bang J. W. (2008). Karyotype analysis and physical mapping using two rRNA genes in dioecious plant, *Humulus japonicus Sieboid* & *Zucc*. Genes. Genomics 30, 157–161.

[B17] Lansky A. J. Costa R. A. Mooney M. Midei M. G. Lui H. K. Strickland W.. (2005). Gender-based outcomes after paclitaxel-eluting stent implantation in patients with coronary artery disease. J. Am. Coll. Cardiol. 45, 1180–1185. 10.1016/j.jacc.2004.10.076 15837246

[B18] Martin F. W. (1966). Sex ratio and sex determination in Dioscorea. J. Hered. 57, 95–99. 10.1093/oxfordjournals.jhered.a107485

[B19] Nadeem M. Rikhari H. Kumar A. Palni L. Nandi S. (2002). Taxol content in the bark of himalayan yew in relation to tree age and sex. Phytochemistry 60, 627–631. 10.1016/S0031-9422(02)00115-2 12126710

[B20] Obbard D. J. Harris S. A. Pannell J. R. (2006). Sexual systems and population genetic structure in an annual plant: testing the metapopulation model. Am. Nat. 167, 354–366. 10.1086/499546 16673344

[B21] Ortiz-Miranda A. S. König P. Kahlert H. Scholz F. Osete-Cortina L. Doménech-Carbó M. T.. (2016). Voltammetric analysis of Pinus needles with physiological, phylogenetic, and forensic applications. Anal. Bioanal. Chem. 408, 4943–4952. 10.1007/s00216-016-9588-7 27173392

[B22] Qu K. Zheng Y. Dai S. Qiao S. Z. (2016). Graphene oxide-polydopamine derived N, S-codoped carbon nanosheets as superior bifunctional electrocatalysts for oxygen reduction and evolution. Nano Energy 19, 373–381. 10.1016/j.nanoen.2015.11.027

[B23] Romano I. Bellitti M. R. Nicolaus B. Lama L. Manca M. C. Pagnotta E.. (2000). Lipid profile: a useful chemotaxonomic marker for classification of a new cyanobacterium in *Spirulina* genus. Phytochemistry 54, 289–294. 10.1016/S0031-9422(00)00090-X 10870183

[B24] Ryu J. H. Messersmith P. B. Lee H. (2018). Polydopamine surface chemistry: a decade of discovery. ACS Appl. Mater. Interfaces 10, 7523–7540. 10.1021/acsami.7b19865 29465221 PMC6320233

[B25] Sharma A. Zinta G. Rana S. Shirko P. (2010). Molecular identification of sex in *Hippophae rhamnoides L*. using isozyme and RAPD markers. Forest. Stud. China 12, 62–66. 10.1007/s11632-010-0012-7

[B26] Wang C.-W. Peng S.-L. Li M.-G. Li W.-J. Lin F.-L. (2006). Review of factors affecting the taxoids content of *Taxus* spp. Acta Ecologica Sinica 26, 1583–1590.

[B27] Xiao C. Fan W. Du S. Liu L. Wang C. Guo M. (2017). A novel glycosylated solution from *Dioscorea zingiberensis* CH wright significantly improves the solvent productivity of *Clostridium beijerinckii*. Bioresour. Technol. 241, 317–324. 10.1016/j.biortech.2017.03.176 28577480

[B28] Yang H. Yin H. Shen Y. Xia G. Zhang B. Wu X. (2016). A more ecological and efficient approach for producing diosgenin from *Dioscorea zingiberensis* tubers via pressurized biphase acid hydrolysis. J. Clean. Prod. 131, 10–19. 10.1016/j.jclepro.2016.05.030

[B29] Yang N. Pan X. Chen G. Sarsaiya S. Yu J. Fan X. (2018). Fermentation engineering for enhanced paclitaxel production by taxus media endophytic fungus MF-5 (*Alternaria* sp.). J. Biobased Mater. Bioenergy 12, 545–550. 10.1166/jbmb.2018.1806

[B30] Yin T. Shu Y. Mei R. Wang J. Cai L. Ding Z. (2018). Diterpenoid alkaloids from aconitum taronense and their chemotaxonomic significance. Biochem. Syst. Ecol. 81, 99–101. 10.1016/j.bse.2018.10.004

[B31] Zhang C. Mei X. Liu L. Yu L. (2000). Enhanced paclitaxel production induced by the combination of elicitors in cell suspension cultures of *Taxus chinensis*. Biotechnol. Lett. 22, 1561–1564. 10.1023/A:1005684901329

